# Efficacy and Safety of Sacubitril/Valsartan Versus Enalapril in the Treatment of Heart Failure With Reduced Ejection Fraction: A Systematic Review and Meta-Analysis

**DOI:** 10.7759/cureus.102656

**Published:** 2026-01-30

**Authors:** Rayan Aljubeh, Saif Almuzainy, Mohamed Lemine, Majed Bseiso, Khaled Kadro, Abdullah Metawa, Rizwan Qaisar

**Affiliations:** 1 College of Medicine, University of Sharjah, Sharjah, ARE; 2 Department of Basic Medical Sciences, College of Medicine, University of Sharjah, Sharjah, ARE

**Keywords:** enalapril, heart failure, meta-analysis, sacubitril/valsartan, systematic review

## Abstract

Heart failure with reduced ejection fraction (HFrEF) remains a leading cause of cardiovascular morbidity and mortality worldwide. The aim of this systematic review and meta-analysis was to evaluate the safety and efficacy of sacubitril/valsartan compared to enalapril in patients with HFrEF. The Preferred Reporting Items for Systematic Reviews and Meta-Analyses (PRISMA) guidelines were followed. We systematically searched PubMed, Scopus, Ovid, Cochrane Library, and ProQuest databases from inception to December 23, 2024. Eligible studies were randomized controlled trials (RCTs) or observational studies published in English, comparing sacubitril/valsartan versus enalapril among adult patients diagnosed with HFrEF. The risk ratios (RRs) and mean differences (MDs) with 95% confidence interval (CI) were computed, and p < 0.05 was considered as a level of significance. Statistical analyses were performed using RevMan (Cochrane Collaboration, London, UK). This study included 10 RCTs and two prospective cohort studies with 11,765 patients (5,879 in the sacubitril/valsartan group and 5,886 in the enalapril group, 45.26 to 69.0 years of age). Sacubitril/valsartan significantly reduced all-cause mortality (RR = 0.85, P = 0.0006), cardiovascular mortality (RR = 0.81, P < 0.0001), and heart failure rehospitalization (RR = 0.68, P = 0.006) compared to enalapril. Hypotension was more frequent with sacubitril/valsartan (RR = 1.54, P < 0.00001), while no significant differences were found for hyperkalemia, angioedema, worsening renal function, or left ventricular ejection fraction (LVEF). Sacubitril/valsartan was associated with lower N-terminal pro-B-type natriuretic peptide (NT-proBNP) levels (MD = -427.50, P = 0.009) and better Kansas City Cardiomyopathy Questionnaire (KCCQ) scores (MD = 1.64, P < 0.00001). Sensitivity analyses confirmed robustness and resolved heterogeneity in several outcomes. Publication bias could not be assessed due to the small number of studies (<10), as funnel plot asymmetry and related tests are unreliable with limited studies. This limitation should be considered when interpreting the results, as undetected publication bias remains possible. Sacubitril/valsartan demonstrates superior efficacy over enalapril in key clinical and patient-reported outcomes. Further research is needed to investigate its long-term safety and effectiveness across diverse patient populations.

## Introduction and background

Heart failure (HF) is a leading cause of both morbidity and mortality, affecting over 64 million people worldwide [1,2]. Heart failure with reduced ejection fraction (HFrEF), defined as a left ventricular ejection fraction (LVEF) of ≤40%, represents a significant public health burden and accounts for approximately 50% of all HF cases [3,4].

For decades, angiotensin-converting enzyme (ACE) inhibitors have played a fundamental role in the management of HFrEF and remain a key component of therapeutic strategies [5,6]. They work by inhibiting the conversion of angiotensin I to angiotensin II, thereby targeting key hormonal pathways involved in the pathophysiology of HF [7].

The PARADIGM-HF trial was a landmark study that evaluated the effectiveness of sacubitril/valsartan, an angiotensin receptor-neprilysin inhibitor (ARNI), compared to enalapril, a traditional ACE inhibitor, in patients with HFrEF. The trial found that sacubitril/valsartan led to a 16% reduction in all-cause mortality (hazard ratio (HR) = 0.84, 95% confidence interval (CI): 0.76 to 0.93, p < 0.001), a 20% reduction in cardiovascular death (HR = 0.80, 95% CI: 0.71 to 0.89, p < 0.001), and a 21% reduction in hospitalization for HF (HR = 0.79, 95% CI: 0.71 to 0.89, p < 0.001) compared to enalapril [8]. Neprilysin, a key peptidase, facilitates the degradation of biologically active peptides that maintain cardiovascular homeostasis. Its inhibition enhances the bioavailability of these peptides, thereby attenuating neurohormonal-driven processes such as vasoconstriction, fluid overload, and adverse cardiac remodeling [9,10]. ARNIs combine neprilysin inhibition, which increases levels of beneficial vasoactive peptides, with angiotensin receptor blockade, and this dual mechanism may be superior to ACE inhibition alone. However, potential risks associated with sacubitril/valsartan include hypotension and higher cost compared to traditional ACE inhibitors, which should be considered when selecting therapy [8,11].

A previous systematic review has addressed this topic [12]. However, the current review was considered necessary because it includes three additional studies published between 2021 and 2024 that compare sacubitril/valsartan and enalapril. Additionally, this review examines a broader range of outcomes, including hypotension, hyperkalemia, angioedema, worsening renal function, change in LVEF from baseline, change in N-terminal pro-B-type natriuretic peptide (NT-proBNP) levels from baseline, and change in the Kansas City Cardiomyopathy Questionnaire (KCCQ) clinical score from baseline, providing a more comprehensive evaluation of the benefits and risks of both treatments. The purpose of this study was to conduct a systematic review and meta-analysis comparing the outcomes of sacubitril/valsartan and enalapril in patients with HFrEF.

## Review

Methodology

We followed the Preferred Reporting Items for Systematic Reviews and Meta-Analyses (PRISMA) statement guidelines during the preparation of this systematic review in reporting our methodology and findings.

Eligibility Criteria

For our systematic review and meta-analysis, we applied the following inclusion criteria: (1) randomized controlled trials (RCTs) or observational studies that investigated the use of sacubitril/valsartan as the intervention, compared to enalapril; (2) adult patients (age ≥18 years) diagnosed with HFrEF; (3) availability of outcome data related to at least one of the following outcomes: all-cause mortality, cardiovascular mortality, HF rehospitalization, hypotension, hyperkalemia, angioedema, worsening renal function, change in LVEF from baseline, change in NT-proBNP levels from baseline, or change in KCCQ clinical score from baseline, all of which were pre-specified before data extraction. We excluded animal studies, articles not written in English, reviews, case reports, case series, editorials, conference abstracts, unpublished studies, and studies without full-text.

Search Strategy

To identify relevant studies, we conducted a comprehensive search in five medical electronic databases through December 23, 2024. The databases searched included PubMed, Scopus, Ovid, Cochrane Library, and ProQuest. The search strategy employed a combination of specific keywords and Medical Subject Headings (MeSH) terms aligned with our study objectives. The search terms included: “angiotensin neprilysin,” “angiotensin receptor-neprilysin inhibitor,” “ARNI,” “sacubitril/valsartan,” “sacubitril valsartan,” “sacubitril and valsartan,” “SV,” “Entresto,” “LCZ696,” “LCZ 696,” “LBQ657,” “enalapril,” “heart failure,” “cardiac failure,” and “myocardial failure.” We ensured all relevant synonyms and variations were captured. When multiple studies reported on the same population, only the most comprehensive or most recent study was included.

Study Selection

Two authors (R.A. and S.A.) independently applied the inclusion and exclusion criteria to all the records. Screening was performed in a two-step process. The first step was to screen titles and abstracts of the retrieved studies for relevance, and in the second step, full texts of potentially eligible studies were reviewed for inclusion. Disagreements were resolved through discussion. Additionally, references of relevant publications were manually reviewed to ensure comprehensive inclusion of all potential studies of interest, identifying any additional relevant research that may not have been retrieved through database searches.

Data Extraction

Four authors independently extracted data using a standardized online data extraction form. Prior to data extraction, the form was pilot tested to ensure clarity and consistency in reporting. Extracted information included the following: (1) study characteristics (study design, population, sample size, type of intervention and comparator, follow-up period, and findings); (2) patient characteristics (age, sex, comorbidities, baseline ejection fraction (EF)); (3) outcomes of interest, categorized as either primary (all-cause mortality, cardiovascular mortality, and HF rehospitalization) or secondary (hypotension, hyperkalemia, angioedema, worsening renal function, change in LVEF, change in NT-proBNP levels, and change in KCCQ clinical score).

Quality Assessment

Two authors independently assessed the methodological quality of the included studies. For RCTs, the Cochrane Risk of Bias (ROB 1) tool was used, evaluating seven domains: random sequence generation, allocation concealment, blinding of participants and personnel, blinding of outcome assessment, incomplete outcome data, selective reporting, and other potential sources of bias. For cohort studies, the Newcastle-Ottawa Scale (NOS) was used to assess selection, comparability, and outcome domains. Studies were classified as low, moderate, or high risk of bias based on the number and severity of methodological concerns.

Handling of Missing Data

When the mean and standard deviation (SD) were not provided in the included studies, we calculated these values using the available data, such as the median, first and third quartiles, range, and sample size, in accordance with the method outlined by Wan et al. [13].

Data Analysis and Synthesis

Continuous variables are presented as mean ± SD, while categorical variables are shown as n (%). Meta-analyses were performed using risk ratio (RR) with 95% CIs for categorical outcomes, and mean differences (MDs) with 95% CIs for continuous outcomes. When appropriate, the number needed to treat (NNT) was calculated from pooled risk differences to facilitate clinical interpretation. Variables reported by two or more studies were pooled. Statistical pooling was conducted using the inverse variance method for both categorical and continuous variables; a p-value <0.05 was considered statistically significant. Heterogeneity was assessed through visual inspection of the forest plots and quantified using I-square and chi-square tests. A substantial heterogeneity was detected if the I² > 50% or chi² p < 0.10, consistent with Cochrane Handbook recommendations. A random-effects model was applied when outcomes showed heterogeneity to account for potential variation in methodology and participant characteristics between studies; otherwise, a fixed-effects model was used. Sensitivity analysis was performed by removing one study at a time. Statistical analyses were conducted using RevMan 5.3 software (Cochrane Collaboration, London, UK).

Publication Bias

It was not possible to assess publication bias due to the relatively small number of included studies (<10), and no visual inspection of funnel plots was conducted [14].

Results

Search Results

**Figure 1:**
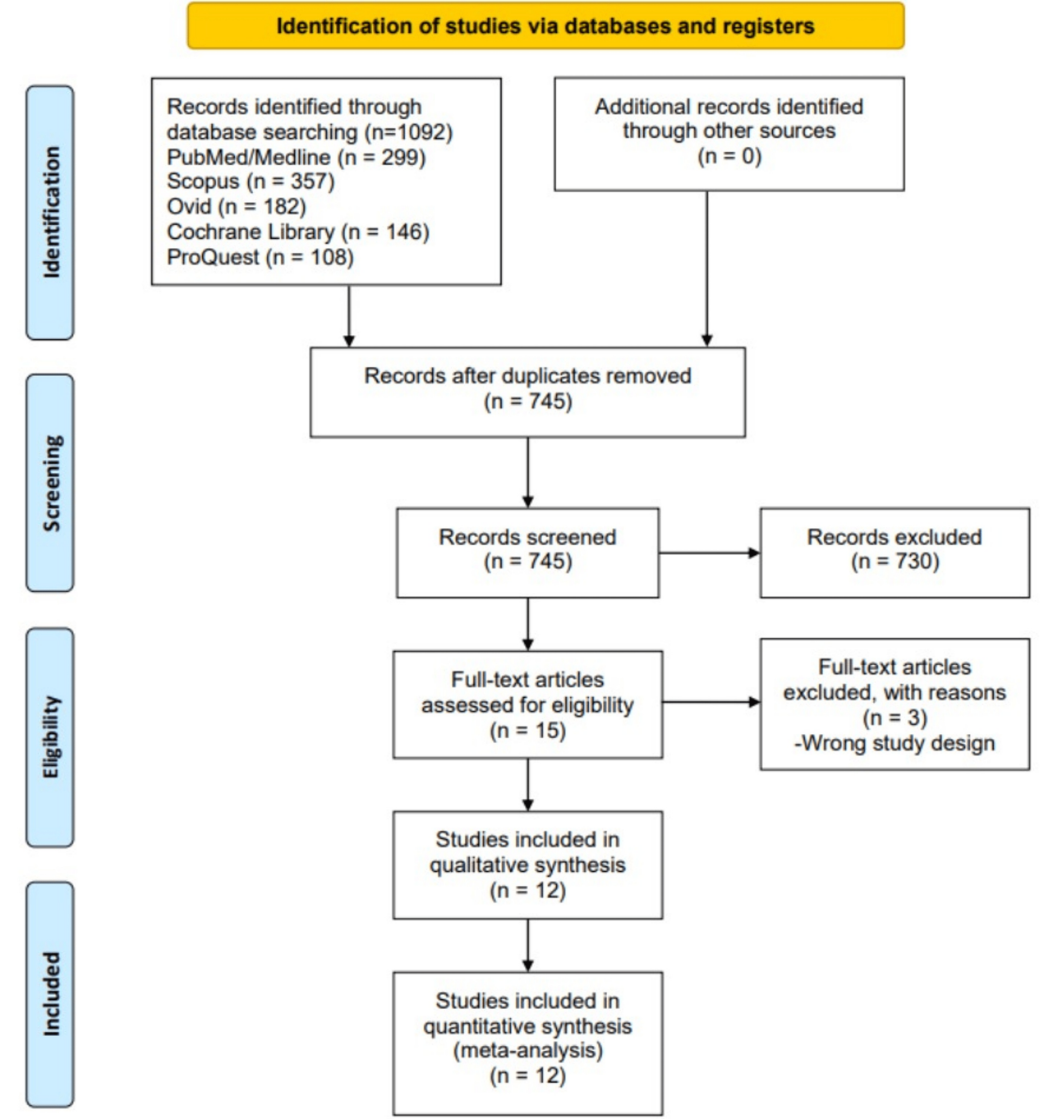
PRISMA flow diagram showing the selection of included studies. PRISMA: Preferred Reporting Items for Systematic Reviews and Meta-Analyses.

Our search yielded 1092 results (Figure 1). After removing duplicates, we screened 745 records and reviewed 15 full-text reports, of which 12 records were included, as shown in Figure 1. Three studies were excluded due to inappropriate study designs, as they were post hoc analyses.

Characteristics of Included Studies and Quality Assessment

In total, 12 studies were included, comprising 11,765 patients (5,879 in the sacubitril/valsartan group and 5,886 in the enalapril group), including 10 RCTs and two prospective cohort studies. Across these, most studies focused on patients with chronic HFrEF (10 studies), while two specifically enrolled patients hospitalized with acute decompensated heart failure (ADHF). This distribution is important when interpreting the pooled estimates, as most evidence reflects outcomes in stable chronic HFrEF populations, with fewer data available for acute settings. Sacubitril/valsartan was compared to enalapril. For more information on characteristics of included studies, see Table 1.

**Table 1:** Characteristics of included studies. HFrEF: heart failure with reduced ejection fraction; NYHA: New York Heart Association; EF: ejection fraction; ADHF: acute decompensated heart failure; S/V: sacubitril/valsartan; 6-MWT: 6-minute walk test; VO₂: volume of oxygen consumed per minute; NT-proBNP: N-terminal pro-B-type natriuretic peptide; HF: heart failure; CV: cardiovascular; PH: pulmonary hypertension; RV-PA: right ventricular–pulmonary artery; TAPSE: tricuspid annular plane systolic excursion; sPAP: systolic pulmonary artery pressure; KCCQ: Kansas City Cardiomyopathy Questionnaire.

Study ID	Study design	Population	Follow-up	Sample size	Intervention	Comparator	Findings
Piepoli 2021 [15]	Randomized controlled trial	Chronic HFrEF, NYHA class II–IV, EF ≤40%	3 months	619 (309 S/V, 310 enalapril)	S/V 200 mg twice daily	Enalapril 10 mg twice daily	S/V showed no significant advantage over enalapril in either the 6-MWT or daytime physical activity as measured by actigraphy.
Santos 2021 [16]	Randomized controlled trial	Chronic HFrEF, NYHA class II–III, EF <40%	5.5 months	52 (29 S/V, 23 enalapril)	S/V 100 mg twice daily	Enalapril 10 or 20 mg twice daily	Compared to enalapril, S/V did not substantially improve peak VO₂ or 6-MWT.
Velazquez 2019 [17]	Randomized controlled trial	Hospitalized for ADHF, EF ≤40%	2 months	881 (440 S/V, 441 enalapril)	S/V 200 mg twice daily	Enalapril 10 mg twice daily	Starting treatment with S/V resulted in a greater decrease in NT-proBNP levels compared to enalapril. The incidence of worsening renal function, hyperkalemia, symptomatic hypotension, and angioedema was similar between the two groups.
Bano 2021 [18]	Randomized controlled trial	Chronic HFrEF, EF <40%	12 months	364 (181 S/V, 183 enalapril)	S/V 50 or 100 mg twice daily	Enalapril 2.5 or 5 mg twice daily	Treatment with S/V was more effective than enalapril in reducing HF-related hospitalizations and deaths, with benefits on CV mortality at least as great as those seen with long-term enalapril use.
Zhao 2022 [19]	Randomized controlled trial	Chronic HFrEF, NYHA class III–IV, EF <40%	6 months	97 (52 S/V, 45 enalapril)	S/V 50 mg twice daily	Enalapril 10 mg once daily	Six months of S/V therapy provided greater benefits than enalapril in patients with HFrEF-induced PH, improving RV-PA coupling, increasing TAPSE, and reducing sPAP.
Desai 2019 [20]	Randomized controlled trial	Chronic HFrEF, NYHA class I–III, EF <40%	3 months	464 (233 S/V, 231 enalapril)	S/V 200 mg twice daily	Enalapril 10 mg twice daily	Treatment with S/V, compared to enalapril, did not lead to a significant reduction in central aortic stiffness.
Tsutsui 2021 [21]	Randomized controlled trial	Chronic HFrEF, NYHA class II-IV, EF ≤35%	33.9 months	225 (113 S/V, 112 enalapril)	S/V 200 mg twice daily	Enalapril 10 mg twice daily	In Japanese patients with HFrEF, S/V and enalapril had similar effects on CV death and HF hospitalization, with S/V being safe and well tolerated.
Halle 2021 [22]	Randomized controlled trial	Chronic HFrEF, NYHA class III, EF ≤40%	3 months	201 (103 S/V, 98 enalapril)	S/V 200 mg twice daily	Enalapril 10 mg twice daily	S/V treatment showed no significant improvement in peak VO₂ compared to enalapril.
McMurray 2014 [8]	Randomized controlled trial	Chronic HFrEF, NYHA class II-IV, EF ≤40%	27 months	8399 (4187 S/V, 4212 enalapril)	S/V 200 mg twice daily	Enalapril 10 mg twice daily	S/V demonstrated greater effectiveness than enalapril in lowering the risks of death and hospitalization due to HF.
Bhat 2022 [23]	Prospective cohort study	Patients with ADHF, NYHA class II-IV, EF <40%	6 months	200 (100 S/V, 100 enalapril)	S/V 200 mg twice daily	Enalapril 10 mg twice daily	The S/V group showed better safety, efficacy, lower mortality, improved echocardiographic outcomes, fewer HF readmissions, and greater KCCQ improvement than the enalapril group.
Zhang 2024 [24]	Prospective cohort study	Chronic HFrEF, NYHA class II-IV, EF <40%	2 months	123 (62 S/V, 61 enalapril)	S/V 200 mg twice daily	Enalapril 5 mg twice daily	S/V was more effective than enalapril in patients with HFrEF failure, improving kidney function, lowering CV markers, and reducing adverse events.
Khandwalla 2020 [25]	Randomized controlled trial	Chronic HFrEF, NYHA class II-III, EF <40%	4 months	140 (70 S/V, 70 enalapril)	S/V 200 mg twice daily	Enalapril 10 mg twice	No significant differences in activity or sleep were found between S/V and enalapril in patients with HFrEF when assessed using a wearable biosensor.

**Figure 2:**
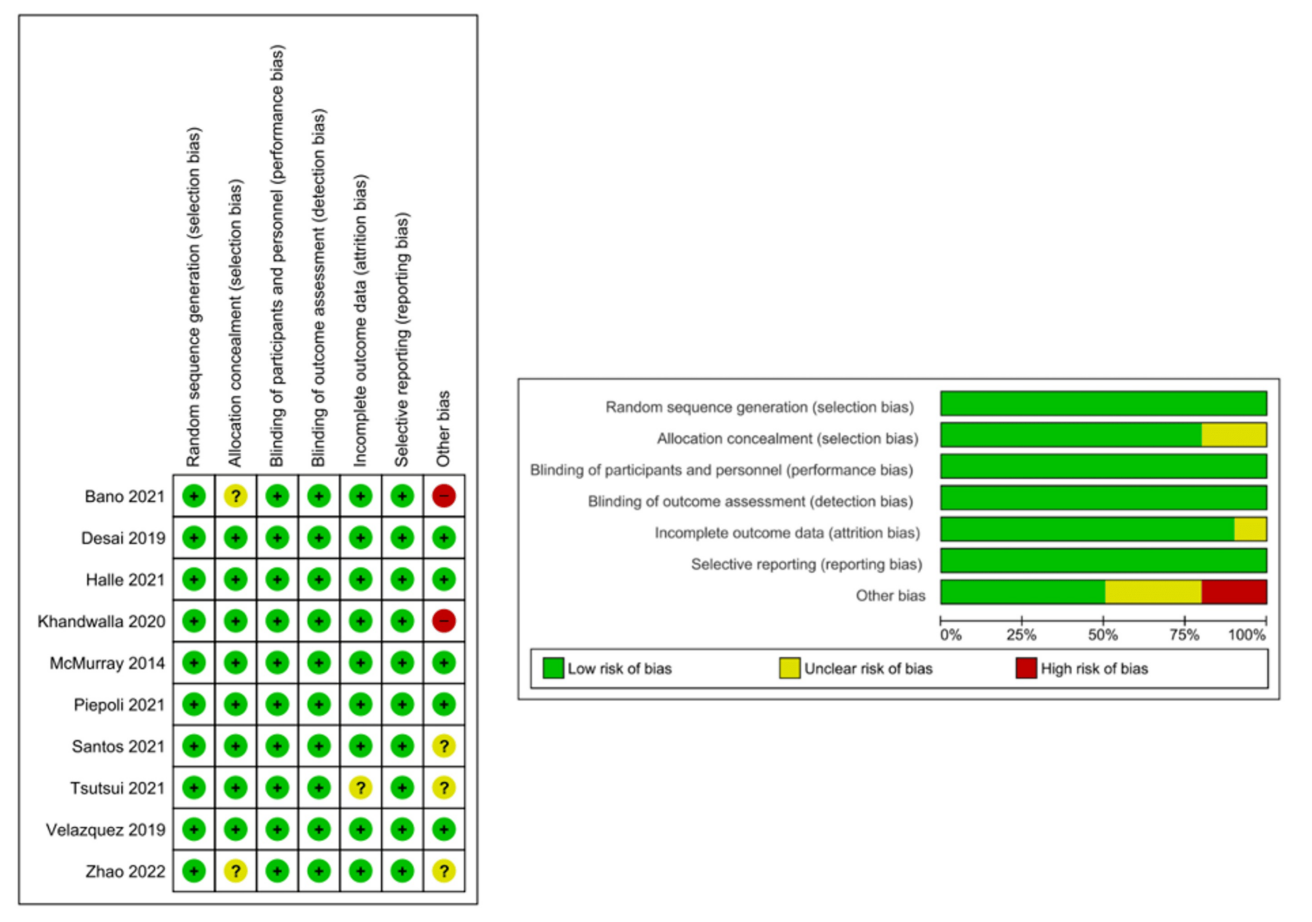
Quality assessment of the included randomized controlled trials using the Cochrane Risk of Bias tool. References [8,15-22,25].

The ROB 1 tool was used for the quality assessment of the RCTs, in which five studies were of high quality [8,15,17,20,22], while three were of moderate quality [16,19,21]. Two studies were of low quality [18,25]. NOS was used for the quality assessment of the cohort studies, in which two studies were of good quality [23,24] (three or four stars in the selection domain, one or two stars in the comparability domain, and two or three stars in the outcome/exposure domain). See Figure 2 and Appendix A for detailed quality assessment scoring.

Characteristics of Included Patients

A pooled analysis of the mean age between the sacubitril/valsartan (61 years and 318 days) and enalapril (62 years and 157 days) groups showed that it was not significantly different between the two groups, indicating balanced randomization and comparable cohorts (MD = -0.25; 95% CI: -1.05 to 0.54; P = 0.53) (Appendix B). Gender was reported in all studies, with pooled analysis showing that the percentage of males was equal between the two groups (RR = 1.01, 95% CI: 0.99 to 1.03, P = 0.22) (Appendix C). The body mass index (BMI) was reported in 10 studies, with a range of 22.13 to 31.2, with pooled results demonstrating no statistically significant difference between the groups (MD = -0.16; 95% CI: -0.36 to 0.04; P = 0.11) (Appendix D). Eight studies reported the heart rate, which was approximately equal between the two groups. Ten studies reported baseline systolic blood pressure (SBP), and seven studies reported baseline diastolic blood pressure (DBP), which were generally equal among the groups. Ten studies reported LVEF, which ranged from 24% to 35.8%. Seven studies reported NT-proBNP in pg/ml, ranging from 560 to 3298.7. Regarding pre-existing conditions, nine studies reported hypertension and diabetes, five studies reported atrial fibrillation (AF), five studies reported hospitalization for HF and ischemic cardiomyopathy, and three studies reported stroke. The presence of pre-existing conditions varied across studies for the two groups. In eight studies, the sacubitril/valsartan group had a higher percentage (123.4%) of pre-existing conditions compared to the enalapril group, which could bias results against sacubitril/valsartan by increasing baseline risk. Conversely, four studies found that the enalapril group had a higher prevalence of these conditions. Several studies reported performing adjusted analyses to account for baseline differences, but the extent and methods of adjustment varied, which should be considered when interpreting outcomes. For detailed baseline characteristics of the included patients, refer to Table 2 and Appendix E.

**Table 2:** Baseline clinical characteristics in the sacubitril/valsartan versus enalapril groups. SD: standard deviation; No.: number of patients; BMI: body mass index; HTN: hypertension; DM: diabetes mellitus; AF: atrial fibrillation; HF: heart failure; NA: not available.

Study ID	Group	Age (mean ± SD)	Male, No. (%)	BMI (mean ± SD)	HTN, No. (%)	DM, No. (%)	AF, No. (%)	Hospitalization for HF, No. (%)	Stroke, No. (%)
Piepoli 2021 [15]	Sacubitril/valsartan	67.16 ± 11.04	238 (77.02)	29.30 ± 4.72	213 (68.93)	96 (31.06)	147 (47.57)	NA	23 (7.44)
	Enalapril	66.62 ± 10.45	249 (80.32)	29.33 ± 4.67	203 (65.48)	177 (37.74)	122 (39.35)	NA	27 (8.71)
Santos 2021 [16]	Sacubitril/valsartan	56.3 ± 10.1	18 (69.2)	28.3 ± 4.7	6 (23.1)	4 (15.4)	1 (3.8)	NA	NA
	Enalapril	61 ± 9.5	14 (73.7)	27.0 ± 4.7	2 (11.1)	3 (16.7)	0 (0.0)	NA	NA
Velazquez 2019 [17]	Sacubitril/valsartan	61 ± 14.9	327 (74.3)	31.2 ± 8.3	329 (74.8)	NA	127 (28.9)	440 (100)	NA
	Enalapril	63 ± 13.4	308 (69.8)	30.7 ± 7.8	309 (70.1)	NA	140 (31.7)	441 (100)	NA
Bano 2021 [18]	Sacubitril/valsartan	53 ± 12	88 (48.62)	NA	165 (91.16)	68 (37.57)	NA	NA	NA
	Enalapril	55 ± 12	90 (49.18)	NA	170 (92.90)	65 (35.53)	NA	NA	NA
Zhao 2022 [19]	Sacubitril/valsartan	68.65 ± 10.48	23 (44.23)	24.66 ± 2.44	32 (61.54)	34 (65.38)	NA	NA	NA
	Enalapril	66.71 ± 10.42	20 (44.44)	25.54 ± 3.30	27 (60.00)	25 (55.56)	NA	NA	NA
Desai 2019 [20]	Sacubitril/valsartan	67.8 ± 9.8	170 (74)	30.0 ± 5.7	NA	NA	NA	128 (55)	NA
	Enalapril	66.7 ± 8.5	185 (79)	30.1 ± 5.8	NA	NA	NA	115 (49)	NA
Tsutsui 2021 [21]	Sacubitril/valsartan	69.0 ± 9.7	96 (86.5)	23.8 ± 4.0	71 (64.0)	52 (46.8)	36 (32.4)	80 (72.1)	11 (9.9)
	Enalapril	66.7 ± 10.9	96 (85.7)	25.1 ± 4.2	82 (73.2)	52 (46.4)	40 (35.7)	82 (73.2)	10 (8.9)
Halle 2021 [22]	Sacubitril/valsartan	66.1 ± 10.8	86 (83.5)	29.2 ± 4.6	NA	NA	NA	57 (55.3)	NA
	Enalapril	67.6 ± 10.0	77 (78.6)	29.6 ± 4.3	NA	NA	NA	51 (52.0)	NA
McMurray 2014 [8]	Sacubitril/valsartan	63.8 ± 11.5	3308 (79)	28.1 ± 5.5	2969 (70.9)	1451 (34.7)	1517 (36.2)	2607 (62.3)	355 (8.5)
	Enalapril	63.8 ± 11.3	3259 (77.4)	28.2 ± 5.5	2971 (70.5)	1456 (34.6)	1574 (37.4)	2667 (63.3)	370 (8.8)
Bhat 2022 [23]	Sacubitril/valsartan	61.2 ± 8.4	70 (70)	NA	55 (55)	31 (31)	NA	NA	NA
	Enalapril	62.6 ± 8.6	74 (74)	NA	47 (47)	33 (33)	NA	NA	NA
Zhang 2024 [24]	Sacubitril/valsartan	46.11 ± 6.79	32 (51.61)	22.13 ± 2.13	15 (24.19)	16 (25.81)	NA	NA	NA
	Enalapril	45.26 ± 5.56	35 (57.38)	22.46 ± 2.55	12 (19.67)	15 (24.59)	NA	NA	NA
Khandwalla 2020 [25]	Sacubitril/valsartan	62.3 ± 8.8	52 (74.3)	28.1 ± 3.8	NA	30 (42.9)	NA	NA	NA
	Enalapril	64.2 ± 11.6	56 (80.0)	28.7 ± 3.9	NA	26 (37.1)	NA	NA	NA

Analysis of Primary Outcomes

**Figure 3:**
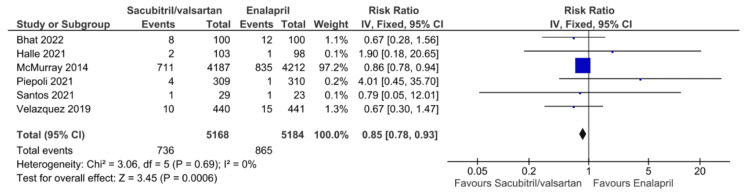
Forest plot of pooled risk ratios of all-cause mortality for sacubitril/valsartan versus enalapril. References [8,15-17,22,23].

**Figure 4:**
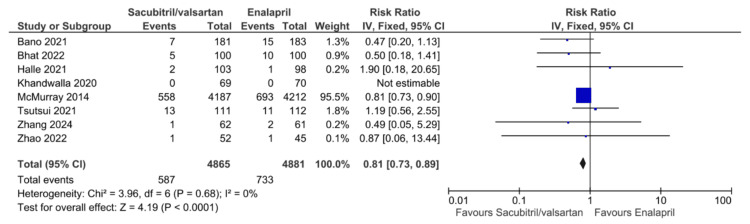
Forest plot of pooled risk ratios of cardiovascular mortality for sacubitril/valsartan versus enalapril. References [8,18,19,21-25].

**Figure 5:**
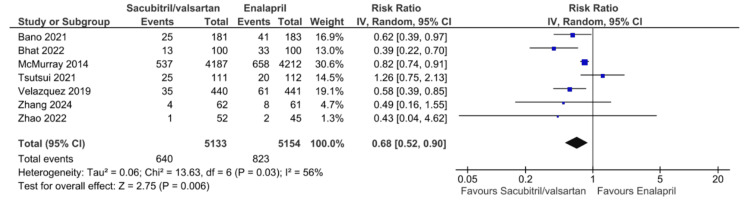
Forest plot of pooled risk ratios of heart failure rehospitalization for sacubitril/valsartan versus enalapril. References [8,17-19,21,23,24].

The primary outcomes of all-cause mortality, cardiovascular mortality, and HF rehospitalization for HFrEF patients taking sacubitril/valsartan compared to enalapril are illustrated in Figures 3-5, respectively. Six studies reported all-cause mortality, with pooled results showing that sacubitril/valsartan significantly reduced all-cause mortality compared to enalapril (RR = 0.85, 95% CI: 0.78 to 0.93, NNT = 40.91, 95% CI: 26.06 to 95.04, P = 0.0006). Eight studies reported cardiovascular mortality, with pooled results showing that sacubitril/valsartan significantly reduced cardiovascular mortality compared to enalapril (RR = 0.81, 95% CI: 0.73 to 0.89, P < 0.0001). Seven studies reported HF rehospitalization, with pooled results showing that sacubitril/valsartan significantly reduced HF rehospitalization compared to enalapril (RR = 0.68, 95% CI: 0.52 to 0.90, P = 0.006). All the above pooled results did not demonstrate significant heterogeneity, except for HF rehospitalization, which showed high heterogeneity (I² = 56%). This heterogeneity may be due to differences in follow-up durations, variations in patient characteristics and disease severity, and differences in study design or definitions of rehospitalization across trials.

Analysis of Secondary Outcomes

**Figure 6:**
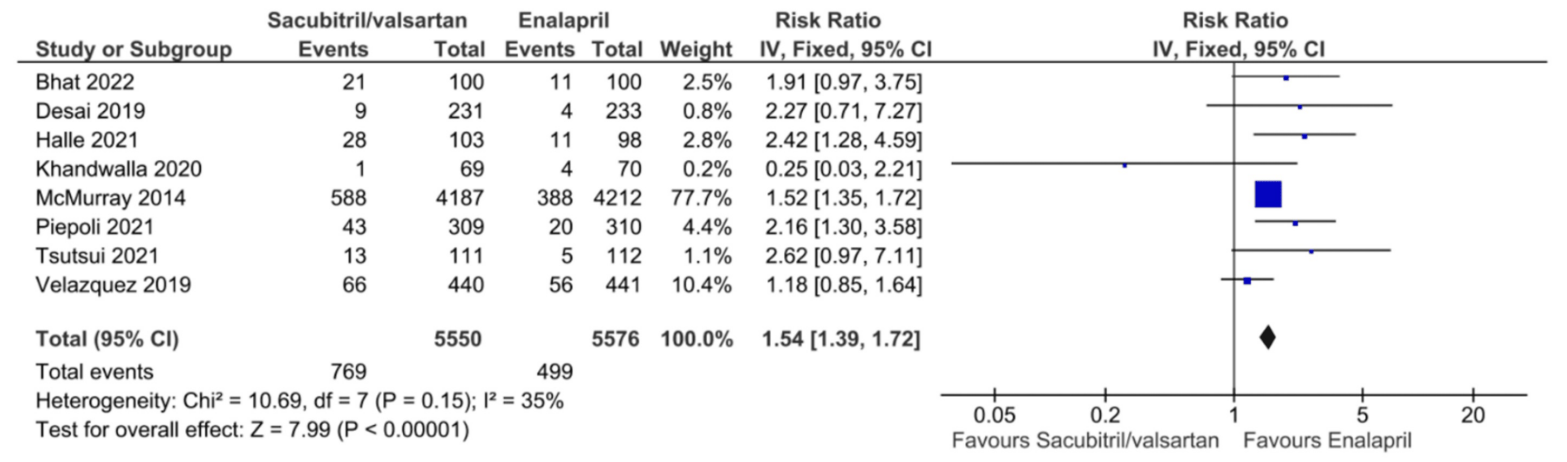
Forest plot of pooled risk ratios of hypotension for sacubitril/valsartan versus enalapril. References [8,15,17,20-23,25].

**Figure 7:**
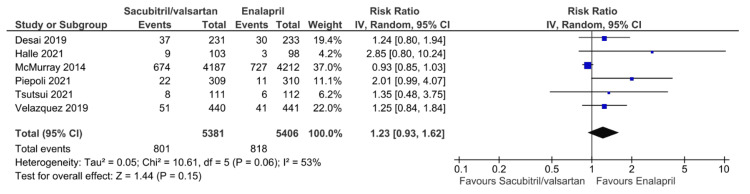
Forest plot of pooled risk ratios of hyperkalemia for sacubitril/valsartan versus enalapril. References [8,15,17,20-22].

**Figure 8:**
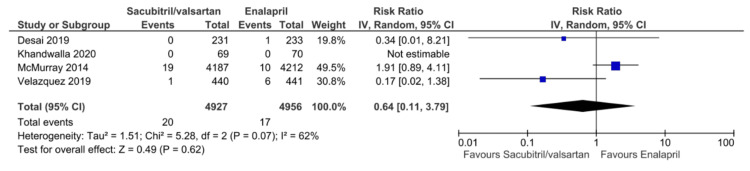
Forest plot of pooled risk ratios of angioedema for sacubitril/valsartan versus enalapril. References [8,17,20,25].

**Figure 9:**
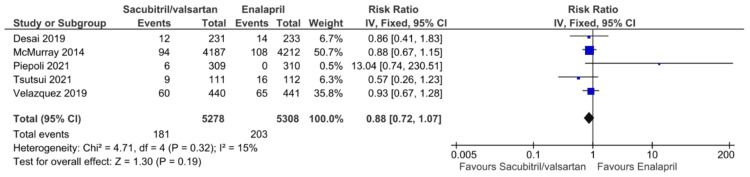
Forest plot of pooled risk ratios of worsening renal function for sacubitril/valsartan versus enalapril. References [8,15,17,20,21].

**Figure 10:**
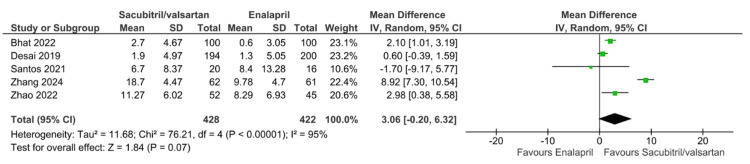
Forest plot of pooled effect estimates comparing change in left ventricular ejection fraction from baseline for sacubitril/valsartan versus enalapril. References [16,19,20,23,24].

**Figure 11:**
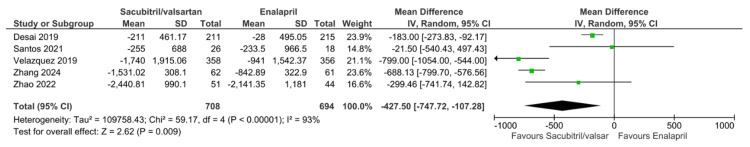
Forest plot of pooled effect estimates comparing change in N-terminal pro-B-type natriuretic peptide from baseline for sacubitril/valsartan versus enalapril. References [16,17,19,20,24].

**Figure 12:**
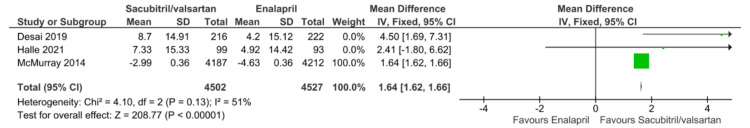
Forest plot of pooled effect estimates comparing change in Kansas City Cardiomyopathy Questionnaire clinical score from baseline for sacubitril/valsartan versus enalapril. References [8,20,22].

All secondary outcomes are displayed in Figures 6-12. Eight studies reported hypotension as a postprocedural complication, with pooled results showing a significantly higher incidence of hypotension with sacubitril/valsartan compared to enalapril (RR = 1.54, 95% CI: 1.39 to 1.72, P < 0.00001). Six studies reported hyperkalemia as a postprocedural complication, with pooled results showing no statistically significant difference between the two groups in hyperkalemia incidence (RR = 1.23, 95% CI: 0.93 to 1.62, P = 0.15). Four studies reported angioedema, with pooled results showing no statistically significant difference between the two groups in angioedema incidence (RR = 0.64, 95% CI: 0.11 to 3.79, P = 0.62). Five studies reported worsening renal function, with pooled results showing no statistically significant difference between the two groups (RR = 0.88, 95% CI: 0.72 to 1.07, P = 0.19). Five studies reported a change in LVEF, with pooled results showing no statistically significant difference between the two groups (MD = 3.06, 95% CI: -0.20 to 6.32, P = 0.07). Five studies reported a change in NT-proBNP, with pooled results showing lower levels of NT-proBNP with sacubitril/valsartan compared to enalapril (MD = -427.50, 95% CI: -747.72 to -107.28, P = 0.009). This consistent trend toward NT-proBNP reduction suggests a potential benefit of sacubitril/valsartan in lowering cardiac stress, supporting further research to confirm its prognostic significance and long-term impact on outcomes. Three studies reported changes in KCCQ scores, with pooled results showing higher scores with sacubitril/valsartan compared to enalapril (MD = 1.64, 95% CI: 1.62 to 1.66, P < 0.00001). All pooled results were highly heterogeneous (I² ≥ 53%), except for hypotension, worsening renal function, and change in KCCQ scores, which showed no heterogeneity.

Sensitivity Analysis

Results of the sensitivity analysis are shown in Appendix F. Removal of Bhat (2022) at leave-one-out analysis resulted in no change in the incidence of HF rehospitalization between sacubitril/valsartan and enalapril, but resolved the heterogeneity. Removal of Piepoli (2021) at leave-one-out analysis resulted in no change in the incidence of hyperkalemia between sacubitril/valsartan as compared to enalapril but resolved the heterogeneity, whereas removing McMurray (2014) at leave-one-out analysis changed the incidence of hyperkalemia so that there is a statistically significant difference with more incidence seen with sacubitril/valsartan as compared with enalapril, but caused no change in the incidence of angioedema between the two groups. In both cases, removing McMurray (2014) resolved the heterogeneity. Removing Velazquez (2019) at leave-one-out analysis caused no change in the incidence of angioedema but resolved the heterogeneity. Removal of Zhang (2024) in the leave-one-out analysis resulted in a significant difference in LVEF, favoring sacubitril/valsartan over enalapril and resolving the heterogeneity.

Publication Bias

It was not possible to assess publication bias due to the relatively small number of included studies (<10) [14].

Discussion

Our systematic review and meta-analysis showed that sacubitril/valsartan significantly reduces all-cause mortality compared to enalapril, with an NNT of 40.91 (95% CI: 26.06 to 95.04). It also significantly reduces cardiovascular mortality and decreases HF rehospitalization. However, sacubitril/valsartan was associated with a higher risk of hypotension. There were no significant differences in hyperkalemia, angioedema, worsening renal function, or LVEF. Reduced NT-proBNP levels with sacubitril/valsartan indicate more effective cardiac performance and a more balanced neurohormonal profile. Improved KCCQ scores suggest better symptomatic control and enhanced quality of life. These findings support the clinical benefits of sacubitril/valsartan in managing patients with HFrEF.

Six studies in this systematic review and meta-analysis confirmed that sacubitril/valsartan is more effective than enalapril in reducing all-cause mortality in patients with EF less than 40%, consistent with the clinical definition of HFrEF, and in line with current American Heart Association/American College of Cardiology (AHA/ACC) guideline recommendations for guideline-directed medical therapy in HFrEF [3]. This supports initiating sacubitril/valsartan early in newly diagnosed patients with EF <40% and in those with prior ACE inhibitors/angiotensin receptor blockers (ARBs) use. These results are in alignment with the PARADIGM-HF trial [8] and the recent Journal of the American College of Cardiology (JACC) Advances meta-analysis [26], both of which demonstrated that the mortality benefit of sacubitril/valsartan is limited to patients with reduced EF. In contrast, large trials such as PARAGON-HF [27], not included in our meta-analysis due to differing populations and EF criteria, reported no significant reduction in all-cause mortality with sacubitril/valsartan. It is important to note that heart failure with preserved ejection fraction (HFpEF) trials vary considerably in their EF thresholds, often using cut-offs of ≥45% or ≥50%, and enroll heterogeneous patient populations with differing comorbidity burdens and pathophysiologic profiles [7,27]. Since EF is a continuous variable, treatment benefit may decline gradually rather than abruptly at a specific EF cut-off, potentially explaining variability in HFpEF trial outcomes [28]. These trials focused on patients with preserved EF or a recent myocardial infarction, suggesting that the mortality benefit of sacubitril/valsartan may be phenotype specific.

Furthermore, these negative trials may have been underpowered. Other contributing factors include design elements of the PARADIGM-HF trial that may affect external validity. Notably, it excluded patients with recent acute coronary syndromes (less than three months), had a low representation of Black participants (5.1%), and excluded individuals with severe pulmonary disease [8,29]. This underrepresentation of racially and ethnically diverse populations limits the generalizability of the findings, particularly given documented differences in HF epidemiology, pathophysiology, and therapeutic response among different racial groups [1,7].

The mechanisms behind sacubitril/valsartan’s mortality benefit in HFrEF are not fully understood but likely relate to the underlying pathophysiology of HF. These mechanisms, which may coexist, could differ at the molecular level between patients with EF below and above 40%, potentially explaining why sacubitril/valsartan confers a greater benefit in reduced EF [28]. Additionally, extracardiac comorbidities gain prognostic importance as the EF rises, underscoring the notion that EF should be considered a continuum, with possible exceptions around the 50-55% range [28].

Pooled data from eight studies showed a consistent 19% relative risk reduction in cardiovascular mortality. These findings align with results from the PARADIGM-HF trial and exhibit minimal heterogeneity, thereby enhancing the reliability and generalizability of our analysis. Mechanistically, sacubitril/valsartan promotes natriuretic peptide signaling via neprilysin inhibition, resulting in vasodilation, natriuresis, reduced sympathetic activity, and attenuation of adverse cardiac remodeling.

Some studies, however, failed to demonstrate a statistically significant mortality benefit. For example, the TRANSITION trial [30] found that in-hospital initiation of sacubitril/valsartan was safe and feasible but did not significantly impact mortality or rehospitalization during its 10-week follow-up. This was likely due to the trial’s focus on safety and tolerability rather than long-term efficacy. Similarly, a recent JACC Advances meta-analysis involving 14 randomized trials (n = 25,167) found no significant reduction in cardiovascular mortality with sacubitril/valsartan compared to ACE inhibitors/ARBs. However, all-cause mortality was improved in patients with an EF of ≤40%. This discrepancy may stem from broader inclusion criteria, heterogeneous HF phenotypes, and varying study designs and follow-up durations.

A key finding from our study is the 32% relative reduction in HF rehospitalization, emphasizing sacubitril/valsartan’s ability to enhance long-term clinical stability and reduce healthcare utilization. This finding is consistent with pooled participant-level data from the PARADIGM-HF and PARAGON-HF trials, which demonstrated an absolute risk reduction of 2.1 hospitalizations per 100 patient-years and an NNT of 48 over a median follow-up of 2.8 years in over 13,000 patients [31]. Recurrent hospitalizations in HFrEF are associated with poorer outcomes and increased mortality risk [31]. The mechanism underlying this reduction may include improved left ventricular function, alleviation of pulmonary hypertension, enhanced blood pressure control, and reduced need for diuretics [8,30].

In summary, sacubitril/valsartan provides robust benefits in patients with HFrEF, including reductions in mortality and rehospitalization, as well as improvements in surrogate endpoints and patient-reported outcomes. These findings support the early initiation of sacubitril/valsartan in appropriately selected patients, aiming to improve both clinical outcomes and healthcare efficiency in the management of HF.

Additionally, eight studies reported a higher incidence of hypotension as an adverse effect of sacubitril/valsartan compared to enalapril. This finding is consistent with prior literature, including the PARADIGM-HF trial and PIONEER-HF trial [17,28]. According to the PARADIGM-HF trial, 16% experienced asymptomatic hypotension, while 11.1% experienced symptomatic hypotension. However, despite that, patients’ outcomes still improved post-intervention, and improvement was more pronounced in those with symptomatic hypotension [32]. The mechanism behind hypotension is attributed to sacubitril/valsartan’s increased vasodilatory effect, since neprilysin inhibition increases the half-life of natriuretic peptides, leading to vasodilation and reduced sympathetic activation [32]. This effect is enhanced when combined with valsartan, which causes a significant reduction in peripheral vascular resistance [32]. Although hypotension is more common with sacubitril/valsartan, most cases are mild to moderate and can often be managed with dose titration, temporary dose reduction, or adjustment of concomitant antihypertensives. However, in a subset of patients, persistent or symptomatic hypotension may necessitate treatment interruption or discontinuation. According to the TRANSITION trial, sacubitril/valsartan treatment was infrequently discontinued solely due to hypotension, as it was generally manageable with close monitoring and timely adjustments [30]. Therefore, while hypotension remains an important tolerability concern, it does not routinely warrant discontinuation when appropriate management strategies are implemented. In conclusion, treatment with sacubitril/valsartan increases the incidence of hypotension in patients with HFrEF compared to enalapril; however, it is not an indication for treatment discontinuation, as the benefits outweigh the risk. Further studies are needed to examine the long-term patient outcomes of those experiencing hypotension and what comorbidities are associated with it.

In this meta-analysis, there was no significant difference in the incidence of hyperkalemia or worsening renal function between sacubitril/valsartan and enalapril treatment. These findings align with prior studies, such as the PARADIGM-HF and PIONEER-HF. In the PARADIGM-HF trial, sacubitril/valsartan caused an initial decline in estimated glomerular filtration rate (eGFR) compared to enalapril; however, long-term renal outcomes were similar between the two interventions. Therefore, this suggests that sacubitril/valsartan intervention can be safely given to HFrEF patients without the risk of renal adverse events, assuming that renal function parameters are closely monitored.

Although single-arm studies, such as PROVE-HF, reported an approximate 9.4% absolute increase in LVEF with sacubitril/valsartan [33], our meta-analysis comparing sacubitril/valsartan to enalapril found no statistically significant difference in LVEF. This may reflect the inclusion of RCTs that were not primarily powered to assess surrogate outcomes, such as LVEF, where reverse remodeling effects may be more modest or less consistently captured.

Furthermore, this analysis revealed that treatment with sacubitril/valsartan was associated with reduced levels of NT-proBNP, a key biomarker of myocardial wall stress and hemodynamic burden. The PIONEER-HF trial supports this finding, demonstrating that NT-proBNP levels decline rapidly following the initiation of sacubitril/valsartan, correlating with improved short-term clinical outcomes. Similarly, findings from the PROVE-HF study further highlight a significant and sustained reduction in NT-proBNP over time with continued sacubitril/valsartan therapy [33]. Lower NT-proBNP concentrations reflect reduced neurohormonal activation and ventricular strain, suggesting a favorable effect on cardiac function and prognosis in patients with HFrEF.

Finally, our results show that sacubitril/valsartan treatment is associated with improved patient-reported KCCQ score compared to enalapril. This is a good indication of better symptomatic control, improved physical function, and lower social barriers. A higher KCCQ score is associated with better quality of life, which is a vital consideration in the long-term management of HF.

This study has several limitations. First, the total number of included studies was relatively small (n = 12), limiting the power to detect publication bias and conduct subgroup analyses. Undetected publication bias could potentially lead to overestimation of treatment effects, thereby skewing clinical interpretation and decision-making. Second, high heterogeneity was observed in several secondary outcomes, including LVEF, NT-proBNP levels, and KCCQ scores, which may be attributed to differences in study design, follow-up duration, population characteristics, and definitions of outcomes. These variations reduce the comparability of results across studies and may limit the extent to which findings can be applied to broader clinical populations. Third, two of the included studies were observational in design, potentially introducing selection and reporting biases. Given this, we recommend that future meta-analyses stratify results by study design to further test the robustness of findings. Fourth, some RCTs were excluded due to differing patient populations (for example, preserved EF), which may limit the generalizability of these findings beyond the HFrEF population. Fifth, differences in baseline characteristics, particularly comorbidities, across studies may have confounded the pooled estimates, despite attempts to balance groups during analysis. While sensitivity analyses using leave-one-out methods supported the robustness of results, we acknowledge that the small number of included studies limited our ability to perform meta-regression and subgroup analyses to further explore heterogeneity, particularly for secondary outcomes with high I² values. Finally, the use of the older Cochrane ROB 1 tool, rather than the updated Cochrane Risk of Bias 2 (ROB 2) tool, represents a methodological limitation, and future studies should consider adopting Cochrane ROB 2 for more comprehensive risk-of-bias assessments. Future research should focus on larger, well-designed RCTs with longer follow-up durations and standardized outcome definitions to improve evidence quality and reduce heterogeneity.

## Conclusions

This review demonstrates that sacubitril/valsartan is superior to enalapril in reducing all-cause mortality, cardiovascular mortality, and HF rehospitalization in patients with HFrEF. Additionally, sacubitril/valsartan was associated with significant improvements in markers such as NT-proBNP levels and KCCQ scores, suggesting enhanced cardiac function and improved quality of life; however, no significant difference was observed in LVEF. Regarding safety, sacubitril/valsartan was associated with a higher incidence of hypotension compared to enalapril. Nevertheless, there were no significant differences in the risks of hyperkalemia, angioedema, or worsening renal function. While the efficacy benefits are clear, these safety considerations highlight the importance of individualized treatment decisions and careful patient monitoring. These findings support the early initiation of sacubitril/valsartan in appropriately selected patients with HFrEF, where the clinical benefits outweigh the risks, especially when managed with appropriate monitoring.

## Figures and Tables

**Figure 13:**
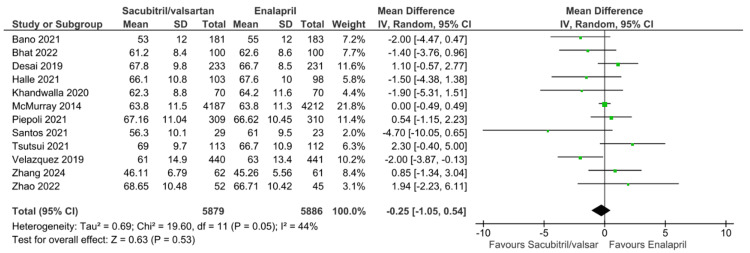
Forest plot of pooled effect estimates comparing the mean age of patients for sacubitril/valsartan versus enalapril. References [8,15-25].

**Figure 14:**
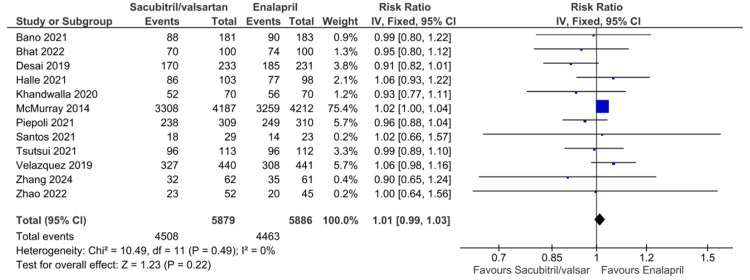
Forest plot of pooled effect estimates comparing the gender (male) proportion of patients for sacubitril/valsartan versus enalapril. References [8,15-25].

**Figure 15:**
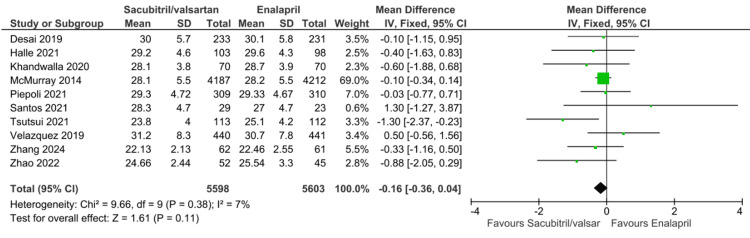
Forest plot of pooled effect estimates comparing the mean body mass index of patients for sacubitril/valsartan versus enalapril. References [8,15-17,19-22,24,25].

**Figure 16:**
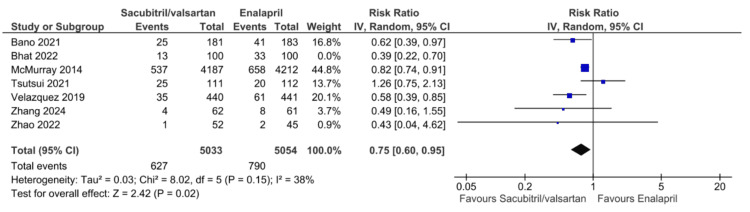
Forest plot showing sensitivity analysis of heart failure rehospitalization after removing Bhat et al. at leave-one-out analysis. References [8,17-19,21,23,24].

**Figure 17:**
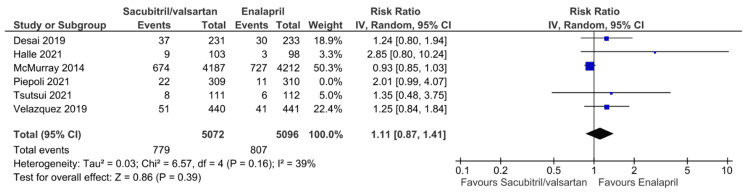
Forest plot showing sensitivity analysis of hyperkalemia after removing Piepoli et al. at leave-one-out analysis. References [8,15,17,20-22].

**Figure 18:**
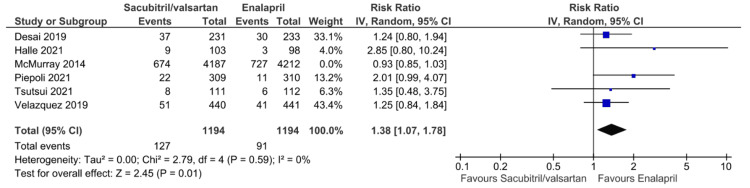
Forest plot showing sensitivity analysis of hyperkalemia after removing McMurray et al. at leave-one-out analysis. References [8,15,17,20-22].

**Figure 19:**
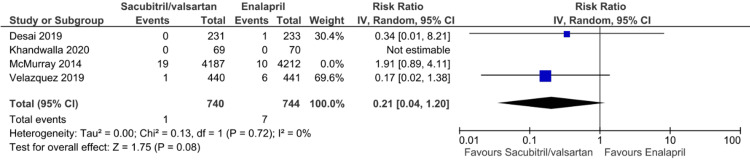
Forest plot showing sensitivity analysis of angioedema after removing McMurray et al. at leave-one-out analysis. References [8,17,20,25].

**Figure 20:**
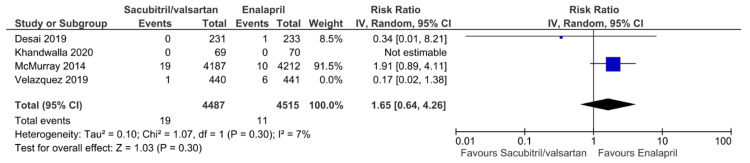
Forest plot showing sensitivity analysis of angioedema after removing Velazquez et al. at leave-one-out analysis. References [8,17,20,25].

**Figure 21:**
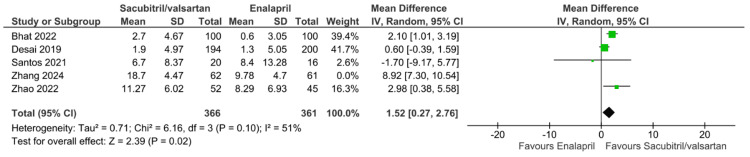
Forest plot showing sensitivity analysis of left ventricular ejection fraction after removing Zhang et al. at leave-one-out analysis. References [16,19,20,23,24].

## Appendices

Appendix A

**Table 3:** Quality assessment of included observational studies using the Newcastle-Ottawa Scale.

Study ID	Selection of cohorts				Comparability of cohorts	Outcome			Score
	Representativeness of the exposed cohort	Selection of the non-exposed cohort	Ascertainment of exposure	Demonstration that the outcome of interest was not present at the start of the study	Comparability of cohorts on the basis of the design or analysis	Assessment of outcome	Was the follow-up long enough for outcomes to occur?	Adequacy of follow-up of cohorts	
Bhat 2022 [23]	-	*	*	*	*	*	*	*	7
Zhang 2024 [24]	-	*	*	*	*	*	-	*	6

Appendix B

Appendix C

Appendix D

Appendix E

**Table 4:** Baseline cardiovascular parameters in the sacubitril/valsartan versus enalapril groups. SD: standard deviation; SBP: systolic blood pressure; DBP: diastolic blood pressure; ICM: ischemic cardiomyopathy; No.: number of patients; LVEF: left ventricular ejection fraction; NT-pro-BNP: N-terminal pro–B-type natriuretic peptide; NYHA: New York Heart Association; NA: not available.

Study ID	Group	Heart rate, beats/min (mean ± SD)	SBP (mean ± SD)	DBP (mean ± SD)	ICM, No. (%)	LVEF, % (mean ± SD)	NT-proBNP, pg/mL (mean ± SD)	NYHA functional class, No. (%)			
								Class I	Class II	Class III	Class IV
Piepoli 2021 [15]	Sacubitril/valsartan	70.81 ± 12.91	126.32 ± 16.45	76.09 ± 10.13	177 (57.28)	NA	NA	NA	161 (52.10)	146 (47.25)	2 (0.65)
	Enalapril	70.22 ± 11.59	126.07 ± 15.84	76.02 ± 10.42	174 (56.13)	NA	NA	NA	162 (52.26)	146 (47.10)	2 (0.65)
Santos 2021 [16]	Sacubitril/valsartan	70 ± 15.6	108.0 ± 16.4	66.7 ± 10.9	NA	27.0 ± 7.0	767.1 ± 687.6	NA	14 (53.8)	12 (46.2)	NA
	Enalapril	70.3 ± 20.5	107.3 ± 20.5	69 ± 11.9	NA	26.0 ± 11.9	1054.5 ± 1188.3	NA	8 (44.4)	10 (55.6)	NA
Velazquez 2019 [17]	Sacubitril/valsartan	88.2 ± 10.2	120.2 ± 17.1	NA	NA	24 ± 8.9	3298.7 ± 2821.1	4 (0.9)	100 (22.7)	283 (64.3)	39 (8.9)
	Enalapril	87.3 ± 8.6	119.7 ± 17.1	NA	NA	25 ± 7.4	2938.7 ± 2643.3	5 (1.1)	122 (27.7)	269 (61)	36 (8.2)
Bano 2021 [18]	Sacubitril/valsartan	81.7 ± 14.9	NA	NA	NA	31.21 ± 4.12	NA	NA	NA	NA	NA
	Enalapril	81.0 ± 14.1	NA	NA	NA	31.82 ± 4.02	NA	NA	NA	NA	NA
Zhao 2022 [19]	Sacubitril/valsartan	NA	133.58 ± 15.27	75.73 ± 9.61	35 (67.31)	35.80 ± 3.94	2899.27 ± 1136.33	NA	NA	28 (53.85)	24 (46.15)
	Enalapril	NA	128.67 ± 16.62	80.09 ± 13.10	29 (64.44)	35.18 ± 4.73	2814.49 ± 1356.44	NA	NA	21 (46.67)	24 (53.33)
Desai 2019 [20]	Sacubitril/valsartan	79.35 ± 12.00	131 ± 15	77 ± 10	137 (59)	34 ± 10	560 ± 307	33(14)	152 (66)	56 (20)	NA
	Enalapril	84.71 ± 20.32	130 ± 13	78 ± 10	146 (63)	33 ± 10	595 ± 294	28(12)	161 (69)	44 (19)	NA
Tsutsui 2021 [21]	Sacubitril/valsartan	68 ± 11	123.6 ± 17.8	72.8 ± 13.2	57 (51.4)	28.6 ± 5.1	837 ± 225.4	4 (3.6)	101 (91.0)	6 (5.4)	NA
	Enalapril	68 ± 12	121.2 ± 14.4	72.8 ± 11.9	49 (43.8)	27.7 ± 5.5	841 ± 269	4 (3.6)	104 (92.9)	4 (3.6)	NA
Halle 2021 [22]	Sacubitril/valsartan	73.9 ± 13.8	128.3 ± 14.4	77.7 ± 9.3	61 (59.2)	31.9 ± 6.1	NA	NA	0 (0)	97 (99.0)	NA
	Enalapril	72.3 ± 12.2	127.6 ± 17.6	77.8 ± 10.0	64 (65.3)	32.0 ± 5.7	NA	NA	1 (1.0)	200 (99.5)	NA
McMurray 2014 [8]	Sacubitril/valsartan	NA	122 ± 15	NA	2506 (59.9)	29.6 ± 6.1	1631 ± 560	180 (4.3)	2998 (71.6)	969 (23.1)	33 (0.8)
	Enalapril	NA	121 ± 15	NA	2530 (60.1)	29.4 ± 6.3	1594 ± 597	209 (5.0)	2921 (69.3)	1049 (24.9)	27 (0.6)
Bhat 2022 [23]	Sacubitril/valsartan	72 ± 12	107.6 ± 9.4	65.3 ± 7.1	NA	NA	NA	0 (0)	36 (36)	47 (47)	17 (17)
	Enalapril	73 ± 12	106.8 ± 10.8	64.3 ± 7.6	NA	NA	NA	0 (0)	38 (38)	42 (42)	20 (20)
Zhang 2024 [24]	Sacubitril/valsartan	NA	NA	NA	NA	33.46 ± 3.89	2832.16 ± 322.44	0 (0)	13 (20.97)	33 (53.23)	16 (25.81)
	Enalapril	NA	NA	NA	NA	33.78 ± 4.12	2760.15 ± 339.15	0 (0)	11 (18.03)	32 (52.46)	18 (29.51)
Khandwalla 2020 [25]	Sacubitril/valsartan	NA	124.0 ± 14.1	NA	NA	31.1 ± 7.9	NA	0 (0)	64 (91.4)	6 (8.6)	0 (0)
	Enalapril	NA	128.5 ± 16.6	NA	NA	30.6 ± 7.7	NA	0 (0)	62 (88.6)	8 (11.4)	0 (0)

Appendix F
